# Evolution of Drug-Resistant *Mycobacterium tuberculosis* Strains and Their Adaptation to the Human Lung Environment

**DOI:** 10.3389/fmicb.2021.612675

**Published:** 2021-02-04

**Authors:** Anna Allué-Guardia, Juan I. García, Jordi B. Torrelles

**Affiliations:** Population Health Program, Tuberculosis Group, Texas Biomedical Research Institute, San Antonio, TX, United States

**Keywords:** *Mycobacterium tuberculosis*, drug resistance, evolution, bacterial–host interactions, next generation sequencing

## Abstract

In the last two decades, multi (MDR), extensively (XDR), extremely (XXDR) and total (TDR) drug-resistant *Mycobacterium tuberculosis* (*M.tb*) strains have emerged as a threat to public health worldwide, stressing the need to develop new tuberculosis (TB) prevention and treatment strategies. It is estimated that in the next 35 years, drug-resistant TB will kill around 75 million people and cost the global economy $16.7 trillion. Indeed, the COVID-19 pandemic alone may contribute with the development of 6.3 million new TB cases due to lack of resources and enforced confinement in TB endemic areas. Evolution of drug-resistant *M.tb* depends on numerous factors, such as bacterial fitness, strain’s genetic background and its capacity to adapt to the surrounding environment, as well as host-specific and environmental factors. Whole-genome transcriptomics and genome-wide association studies in recent years have shed some insights into the complexity of *M.tb* drug resistance and have provided a better understanding of its underlying molecular mechanisms. In this review, we will discuss *M.tb* phenotypic and genotypic changes driving resistance, including changes in cell envelope components, as well as recently described intrinsic and extrinsic factors promoting resistance emergence and transmission. We will further explore how drug-resistant *M.tb* adapts differently than drug-susceptible strains to the lung environment at the cellular level, modulating *M.tb*–host interactions and disease outcome, and novel next generation sequencing (NGS) strategies to study drug-resistant TB.

## Introduction

Tuberculosis (TB) kills one person every 21s, with ∼10 million cases and ∼1.5 million attributed deaths in 2018 (WHO, 2019). Caused by airborne pathogen *Mycobacterium tuberculosis* (*M.tb*), TB is the top disease killer worldwide due to a single infectious agent. After inhalation, *M.tb* reaches the alveolar space and is bathed in alveolar lining fluid (ALF), being in intimate contact with soluble components of the lung mucosa before interacting with the cellular compartment, including alveolar macrophages (AM) and other immune cells (Arcos et al., 2011; Torrelles and Schlesinger, 2017). This *M.tb*–host interplay during the initial stages of infection will determine TB disease outcome, driving clearance or progression to active (pulmonary or extrapulmonary TB) or latent TB, although the exact bacterial/host determinants and mechanisms that promote a successful infection are still poorly understood. The success of *M.tb* in establishing infection and evading the host immune system is in part due to its unique and dynamic cell envelope, composed mainly of lipids and carbohydrates, which changes to adapt to the different lung microenvironments in response to local environmental cues, and also protects the pathogen against harsh environments and many antimicrobial drugs due to its low permeability (Garcia-Vilanova et al., 2019; Maitra et al., 2019). And thus, most combined treatments for active TB work disrupting the mycobacterial cell envelope.

Standard treatment for drug-susceptible TB consists of administering four first-line drugs for 6 months (2 months of isoniazid, rifampicin, ethambutol and pyrazinamide, followed by 4 months of isoniazid and rifampicin), with a success rate of 85% (WHO, 2019). However, the worldwide emergence of drug-resistant strains in recent years, especially in TB endemic areas (Ellner, 2008; Lange et al., 2019), poses a global threat and compromises the goal of the End TB Strategy of reducing 90% the TB cases by 2035. Different causes have led to increased drug resistance, including insufficient healthcare infrastructure, prescription of the wrong treatment (either wrong dose or treatment length), poor quality drugs or drug unavailability, poor adherence to therapy, or *M.tb* re-infection, among others (Zaman, 2010). Indeed, in 2018, approximately half a million people developed drug-resistant TB (DR-TB) and of these, 78% were multidrug-resistant (MDR-TB), and only one in three confirmed cases were enrolled in treatment (WHO, 2019). Different categories of drug-resistant *M.tb* strains have been defined by the WHO: multi (MDR), extensively (XDR), and extremely (XXDR) drug-resistant TB. MDR-TB, which represent a 3.4% of new TB cases worldwide, are resistant to at least the two first-line drugs isoniazid and rifampicin, and require 9–20 months of costly treatment using second-line drugs, with decreased success rate of 56% when compared to susceptible TB (WHO, 2019). XDR-TB are also resistant to rifampicin and isoniazid, plus fluoroquinolone and at least one of the three injectable second-line drugs (amikacin, kanamycin, or capreomycin), with even a lower treatment success rate of 39% (WHO, 2019). Lastly, XXDR strains are resistant to all first and second-line drugs. More recently, a new category of *M.tb* strains not yet recognized by the WHO have been identified in Italy, India and Iran (Velayati et al., 2009b; Loewenberg, 2012), and named totally drug-resistant TB (TDR-TB) for being resistant to all tested antibiotics plus some of the ones currently in the discovery pipeline (Loewenberg, 2012; Parida et al., 2015). The emergence of these potentially incurable strains stresses the urgent need to develop new drug regimens and/or alternative anti-TB strategies to combat these superbugs (Lange et al., 2018).

Only a few studies have explored the cell envelope composition of drug-resistant *M.tb* strains and their metabolic responses and adaptation to the different environmental pressures during the course of pulmonary infection, which makes it challenging to develop new and effective treatments (Velayati et al., 2009b). Due to an altered cell envelope composition of drug-resistant isolates (Velayati et al., 2013), one can hypothesize that drug-susceptible and drug-resistant strains will adapt differently to the lung microenvironments, driving different infection outcomes. In recent years, new developments in next generation sequencing (NGS) technologies have allowed investigators to gain further insight not only in specific genetic determinants of drug resistance but have also shed some light on how particular *M.tb* strains adapt and interact with the host during infection through genome-wide transcriptomics, which is key in finding novel bacterial determinants that can be targeted in the development of new anti-TB drugs or host-directed therapies (Jeanes and O’Grady, 2016).

In this review, we will discuss different factors driving the emergence of drug-resistant *M.tb* strains, as well as what is known about the adaptation of MDR strains to the different lung environments encountered during the course of infection, and describe recent NGS strategies that could be used to gain a better understanding on *M.tb*–host interactions and key determinants involved in MDR evolution.

## Origin and Evolution of Drug Resistance

*Mycobacterium tuberculosis* is a highly specialized human intracellular pathogen with an extremely conserved genome driving a paradigm of a near-perfect host-pathogen relationship (Brites and Gagneux, 2015). Currently, phylogenetic inferences reveal that there are seven global lineages of *M.tb* strains that have co-evolved with human populations under sympatric and allopatric host-pathogen combinations (Gagneux et al., 2006a; Al-Saeedi and Al-Hajoj, 2017). These *M.tb* strain lineages differ in their geographic distribution, biological fitness, virulence and their propensity to acquire drug resistance; specifically, lineages 2 to 4 have been associated with greater disease burden and drug resistance compared to ancient lineages, e.g., lineages 1, 5, and 6 (Al-Saeedi and Al-Hajoj, 2017; Nguyen et al., 2018).

The emergence of drug resistance in *M.tb* can be divided in two main factors and their interactions: extrinsic factors, which are related to social determinants of TB in populations and the quality of TB control and prevention services; and intrinsic factors, accounting for those related to the acquisition of genetic mutations in drug resistance-associated genes (Gygli et al., 2017; Nguyen et al., 2018; Swain et al., 2020). Regarding the intrinsic factors, mutations in genes coding for drug targets or drug activating enzymes are the primary mode of drug resistance, and they arise mainly through single nucleotide polymorphisms (SNPs) and insertion-deletions (indels). Unlike other bacterial pathogens, acquisition of drug resistance through horizontal gene transfer is not consistently reported in *M.tb* (Namouchi et al., 2012; Dookie et al., 2018). In addition, drug resistance in *M.tb* is commonly believed to be caused by single-step chromosomal mutations. However, there are now evidences suggesting that, at least for certain anti-TB drugs, acquired drug resistance is the result of a stepwise acquisition and fixation of mutations leading to a gradual increase in resistance, initiated with the acquisition of isoniazid resistance, subsequently followed by rifampicin or ethambutol resistance (Cohen et al., 2015). Indeed, resistance to isoniazid through the *KatG*S315T mutation is a common pattern that precedes rifampicin resistance and is conserved globally regardless of the *M.tb* lineage, geographic region and/or time (Manson et al., 2017). DR-TB can occur as primary drug resistance, when a person is directly infected by a drug-resistant *M.tb* strain, or as secondary or acquired drug resistance, occurring due to the acquisition of resistance-conferring mutations during failed treatment of drug-susceptible TB (Dookie et al., 2018). The latter is likely associated to *M.tb* metabolic adaptation to the host lung environment.

In addition to resistance caused by target mutations, several distinctive mechanisms of anti-*M.tb* innate resistance have been also described (Singh et al., 2020). These include: drug accessibility to the target due to the low permeability of the *M.tb* cell envelope, modification of drugs by *M.tb* enzymes, existence of *M.tb* efflux pumps removing out drugs that were able to cross the *M.tb* cell envelope, *M.tb* modulation of its gene expression to adapt to the drug’s effects (or to its presence), and a phenotypic drug tolerance linked to a state of slow growth rate and metabolic shut down. In this regard, during *M.tb* infection certain bacterial subpopulations, known as persisters, can become phenotypically tolerant to antimycobacterial drugs without acquiring genetic mutations (Kester and Fortune, 2014). This reversible phenomenon is usually induced by external stresses such as hypoxia or drug treatment, among others factors (Vilcheze and Jacobs, 2019), and is associated with a nonreplicating status of the bacilli. Several *M.tb* factors and metabolic traits are linked to *M.tb* persistence (Keren et al., 2011; Lee et al., 2019), although the evidence suggests that persistence consists of an array of heterogenous physiological states and mechanisms (Hartman et al., 2017). Mechanisms of *M.tb* persistence are still being elucidated; however, different bacterial metabolic status during early stages of the infection might explain why some *M.tb* subpopulations can become phenotypically drug-resistant.

Indeed, a novel genetically encoded mechanism that causes reversible drug tolerance was recently described in *M.tb* (Bellerose et al., 2019; Safi et al., 2019; Vargas and Farhat, 2020). This phase variation phenomenon is caused by transient frameshift mutations in a 5’ homopolymeric region in the *glpK* gene, which encodes the glycerol-3-kynase required for *M.tb* glycerol catabolism. This phenomenon has been observed in several clinical isolates which, as a consequence, generate bacterial variants (small smooth colonies) that exhibit a drug-tolerant phenotype easily reversible through the introduction of additional insertions/deletions in the same *glpK* region. This frameshift mutations are particularly enriched in MDR- and XDR-*M.tb* strains, and suggest that variation in GlpK might contribute to the development of drug resistance. Thus, limited efficacy of current TB treatments suggests that heterogeneity of both host and mycobacterial physiologies in the different lung compartments during the different stages of the infection can influence the emergence of persisters and ultimately, increased drug resistance (Warner and Mizrahi, 2006; Ben-Kahla and Al-Hajoj, 2016; Boldrin et al., 2020). Novel antimycobacterial drugs that target persisters populations are critical in order to shorten TB treatment and decrease the emergence and transmission of MDR-*M.tb* strains (Zhang et al., 2012; Mandal et al., 2019). Conversely, mixed clonal infections and/or genetic heterogeneity of *M.tb* populations with different drug susceptibility profiles within a patient can also lead to disparate responses during treatment, promoting drug resistance (Liu et al., 2015).

### Factors Contributing to the Emergence of Drug Resistance

The mechanisms and pathways that result in the emergence and subsequent fixation of *M.tb* resistant strains and its dynamics are not fully understood. However, evidence suggest an interplay of several mechanisms involved during drug selection pressure, including clonal interference, mutation rates, efflux pumps, compensatory mutations, and epistasis (Al-Saeedi and Al-Hajoj, 2017). Exposure of *M.tb* to drugs induces a bacterial stress response while exerting a drug selection pressure; thus, only those *M.tb* strains able to adapt will prevail, initiating a competitive selection process between *M.tb* clones that may acquire different beneficial mutations to survive (clonal interference). In this regard, *M.tb* strain lineages can present different mutation rates and different capacity to acquire drug resistance (Ford et al., 2013), where clones of the Beijing/East Asian lineage (lineage 2) are associated to an hypermutability phenotype mediated through polymorphisms in anti-mutator genes (*mut)* involved in DNA repair systems (Ebrahimi-Rad et al., 2003). Recently, DNA methylation has been proposed as a mechanism for phenotypic plasticity in *M.tb*, aiding rapid adaptation to changing environmental pressures, with heterogeneous DNA methylation reported in MTBC clinical isolates (Modlin et al., 2020), which could have potential implications in the development of drug resistance. Further, because of the drug-induced stress response of *M.tb*, *M.tb* efflux pumps are rapidly upregulated within hours after drug exposure, being responsible of conferring a low-level resistance profile (described in detail below). Interestingly, efflux pump inhibitors reduce the minimum inhibitory concentrations (MICs) of key drugs such as isoniazid, rifampicin, linezolid and some fluoroquinolones (Escribano et al., 2007; Machado et al., 2012). Thus, this efflux pump mediated low-level resistance confers a selective advantage and allows *M.tb* to survive and replicate under sub-optimal drug concentrations, until further development of classical resistance-associated mutations that confer clinical drug-resistant phenotypes (Fonseca et al., 2015; Dheda et al., 2017).

Although beneficial against anti-TB treatment, drug resistance mutations may also impose a fitness cost to *M.tb* survival, as they can compromise genes involved in essential mycobacterial cell functions. This subject is a matter of debate aiming to elucidate the mechanisms involved in impaired fitness, transmissibility and virulence of DR-*M.tb* strains. The concept of reduced virulence of MDR-*M.tb* strains comes from early experiments in the Guinea pig infection model (Middlebrook and Cohn, 1953). More recently, clinical isolates with common mutations for isoniazid, rifampicin, and streptomycin were associated with low or absent *in vivo* fitness cost (Comas et al., 2011). Thus, clinical isolates undergo mutations to overcome fitness deficits associated to drug resistance, setting the basis for the compensatory evolutionary role in the spread of DR-TB (Nguyen et al., 2018). There is a wide diversity of resistance-conferring mutations in *M.tb*; however, during selective pressure, specific mutations in clinical isolates are associated with a high-level of resistance without losing fitness or transmissibility (Casali et al., 2014). Different compensatory mutations were demonstrated for *rpoA* and *rpoC* in rifampicin-resistant *rpoB* mutants (Comas et al., 2011; de Vos et al., 2013), for *ahpC* in isoniazid-resistant *KatG* mutants (Sherman et al., 1996), and 16S RNA for aminoglycoside resistance (Shcherbakov et al., 2010). This evidence supports the successful expansion of MDR and XDR-*M.tb* strains worldwide (Merker et al., 2018; Cohen et al., 2019a).

Epistasis, a form of interaction between genes or mutations that influences a phenotype, is also thought to drive the evolution of DR-TB (Borrell et al., 2013; Koch et al., 2014). In fact, some *M.tb* compensatory mutations are associated with epistatic interactions. Epistasis is conceptually linked to fitness and is divided into negative and positive epistasis; the latter meaning that the interaction between genes or mutations has a smaller fitness cost compared to the genetic determinants alone. Different positive and negative epistasis exist in *M.tb* between drug-resistant mutations and between drug resistance-associated mutations and compensatory mutations (Phillips, 2008; Nguyen et al., 2018). In this context, different lineages of *M.tb* carrying identical rifampicin-associated mutations showed different levels of fitness cost, supporting the idea that epistasis is influenced by *M.tb* strain’s genetic background (Gagneux et al., 2006b; Borrell and Gagneux, 2011; Li et al., 2017). Epistatic interactions may also determine the order in which drug resistance mutations and compensatory mutations arise, playing a key role in defining the evolutionary processes toward acquisition of MDR-TB (Salverda et al., 2011; Muller et al., 2013).

## Adaptation of Drug-Resistant *M.tb* Strains to the Human Lung Environment

**FIGURE 1:**
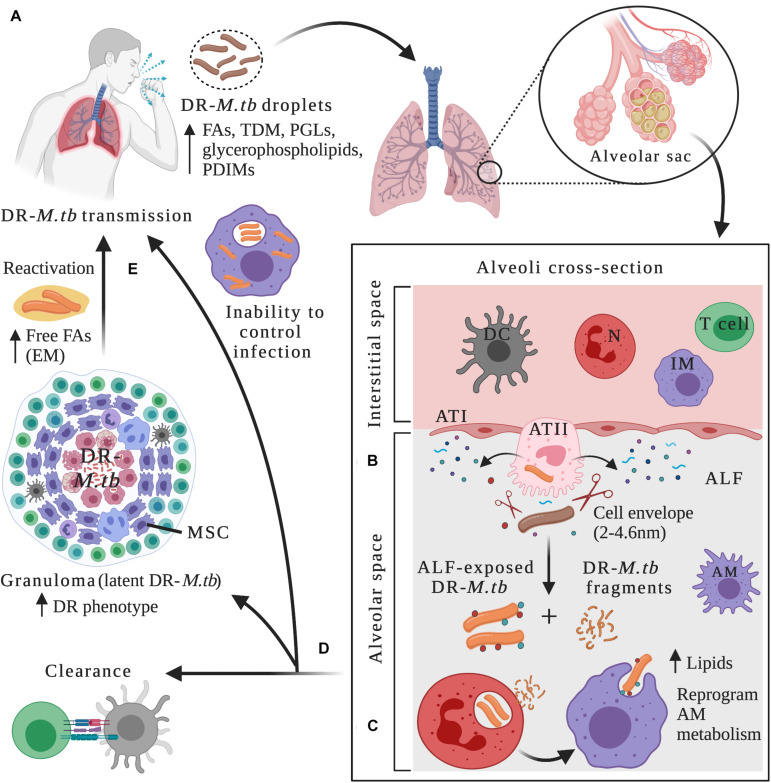
Drug-resistant *M.tb*–host interactions within lung microenvironments at different stages of the infection. **(A)** After being in close contact with an individual with active TB disease, infection can be initiated through inhalation of drug-resistant (DR) *M.tb*-containing droplets. DR-*M.tb* bacilli contain altered levels of cell envelope lipids such as free fatty acids (FAs), trehalose dimycolate (TDM), Phthiocerol dimycocerosates (PDIMs), phenolic glycolipids (PGLs) and glycerophospholipids, among others. Upon bypassing upper respiratory tract barriers, DR-*M.tb* will ultimately reach the alveoli, a sac-like structure composed of a thin layer of alveolar epithelial cells type I (ATIs, with structural and gas exchange function) and alveolar epithelial cells type II (ATII, with secretor function) surrounded by capillaries. Alveolar macrophages (AMs) are resident phagocytes that populate the alveolar space, while the interstitial space surrounding the alveoli contains interstitial macrophages (IMs), dendritic cells (DCs), neutrophils (N), and T cells, among other host cells. **(B)** In the alveolar space, DR-*M.tb* bacilli first interact with host soluble innate components present in the alveolar lining fluid (ALF), where hydrolases (represented as scissors) can cleave and modify the *M.tb* cell envelope, releasing cell envelope fragments into the alveolar space. **(C)** Subsequently, ALF-modified DR-*M.tb* bacilli will interact with AMs (professional phagocytes) and/or with ATs (non-professional phagocytes), as well as with other host innate immune cells (e.g., neutrophils, DCs). Released DR-*M.tb* fragments are immunogenic and could attract neutrophils to the infection site driving local oxidative stress and inflammation, which could assist resident resting AMs to clear the infection. **(D)** The outcome of these initial interactions will resolve in ALF-exposed DR-*M.tb* clearance, establishment of a successful infection driving primary active TB disease, or a latent *M.tb* infection defined by *M.tb* persisters within granulomas, a niche that provide a protective environment against anti-TB drugs, thus increasing the DR-*M.tb* phenotype. Surrounding mesenchymal stem cells (MSCs) can also dampen immune responses and provide a protective intracellular environment for *M.tb* persistence. **(E)** Reactivation and subsequent progression to active TB disease can happen when granulomas fail to contain DR-*M.tb*, with extracellular DR-*M.tb* growth that leads to lung tissue destruction and cavity formation. It has been suggested that in this scenario, DR-*M.tb* secretes free fatty acids, creating some kind of extracellular matrix (EM) that further shields the DR-*M.tb* against TB drugs. Figure created with BioRender.com.

*Mycobacterium tuberculosis* evolved from a mycobacterial ancestor to effectively infect and persist in the human host (Brites and Gagneux, 2015; Jia et al., 2017). Its higher hydrophobicity compared to other mycobacterial species might have contributed to increased transmission through aerosolization (Jankute et al., 2017). After being inhaled and during the course of infection, *M.tb* gets in contact with very different host lung microenvironments, which can be extracellular (during the initial stages of infection and before interacting with alveolar phagocytes and other immune cells, after escaping necrotic cells, or after re-activation in cavities); or intracellular (in alveolar phagocytes such as alveolar macrophages (AMs) during primary infection, or within AMs in granulomas during the latency stage) (Figure 1). In these different environments, *M.tb* has evolved to use host resources to its advantage and adapt its metabolism in order to evade the host immune system, survive, and establish a successful active or latent infection.

The *M.tb* cell envelope provides structural support and protection to osmotic changes, and is composed of four main layers: an inner plasma membrane and periplasmic space, a cell envelope core composed of peptidoglycan (PG) covalently linked to arabinogalactan (AG) and mycolic acids (MAs), the peripheral lipid layer [formed by non-covalently linked lipids and glycolipids such as trehalose dimycolate (TDM), phthiocerol dimycocerosates (PDIMs), mannose-capped lipoarabinomannan (ManLAM), sulfolipids (SLs), phosphatidyl-*myo*-inositol mannosides (PIMs), and phenolic glycolipids (PGLs), among others], and the outermost layer (also named capsule) (Kalscheuer et al., 2019). Although lipids and carbohydrates constitute ∼80% of the cell wall, proteins are also important components of the *M.tb* envelope, being studied as potential drug targets (Gu et al., 2003; Mawuenyega et al., 2005; Niederweis et al., 2010; Chiaradia et al., 2017; Hermann et al., 2020). Several described proteins regulate the permeability of the *M.tb* cell envelope, with important implications in drug resistance. Among many, we find mycobacterial membrane protein Large 3 (MmpL3), Rv3143/Rv1524 axis, and decaprenylphosphoryl-*D*-ribose 2′-epimerase (DprE), which are involved in the export and synthesis of the *M.tb* cell wall, regulating its permeability (Dong et al., 2020). Other *M.tb* cell wall proteins act directly inactivating the drug. This is the case of Rv2170, a putative acetyltransferase that acetylates isoniazid (INH), inducing its breakdown into acetylhydrazine and isonicotinic acid, thus defining a mechanism by which some *M.tb* strains could bypass INH toxicity (Arun et al., 2020).

Besides providing structural support, the *M.tb* cell envelope also plays a critical immunomodulatory role in the bacterium-host crosstalk, where several cell envelope outer molecules are known to participate in different stages of the infection with key implications in *M.tb* pathogenesis and development of drug resistance (Sonawane et al., 2012; Gago et al., 2018; Garcia-Vilanova et al., 2019; Kalscheuer et al., 2019). Indeed, the mycobacterial cell envelope is considered a highly dynamic structure, tightly regulated and remodeled in response to changing host environmental pressures. Consequently, a wide range of cell envelope compositions in different bacterial cells can be found during the course of infection (Kalscheuer et al., 2019). These will determine how *M.tb* interacts with the host immune system and its potential implications for antibiotic resistance (Dulberger et al., 2020).

However, little is known about the temporal changes of the *M.tb* cell envelope in response to local environmental cues and how these can lead to different disease outcomes, especially in DR-*M.tb* strains, since most of the data come from *in vitro* studies and/or specific stages of infection, not necessarily reflecting the actual temporal dynamics of this complex and heterogeneous structure. *M.tb*–host interactions and subsequent infection outcome might also be influenced by *M.tb* metabolic and/or physiologic status before and/or during infection. Below we will discuss recent developments on MDR-*M.tb* adaptation to the different human lung microenvironments, and how the dynamics of the *M.tb* cell envelope might determine disease outcome during the *M.tb*-host interplay.

### Initial *M.tb*–Host Interactions Within the Lung Mucosa

*Mycobacterium tuberculosis* infection requires close contact with an infectious individual with active TB, occurring through airborne transmission of droplets containing viable bacteria (Figure 1A). Infection in the lungs is initiated when the pathogen is inhaled and bypasses bacterial clearance mechanisms present in the first interaction points that act as barriers, including nose and sinuses, tracheobronchial tree and bronchioles, ultimately reaching the alveoli (Torrelles and Schlesinger, 2017) (Figure 1A). In the alveolar space, *M.tb* first comes in close contact with soluble elements present in the lung alveolar mucosa, which is composed of surfactant lipids and a hypophase layer or ALF (Figure 1B). ALF contains surfactant proteins, hydrolytic enzymes, and complement proteins, among others, all critical components of the host soluble innate immune response. Indeed, ALF hydrolases are known to interact and modify the cell envelope of *M.tb*, significantly reducing the amount of two major virulence factors, ManLAM and TDM on the bacterial cell envelope surface (Arcos et al., 2011) (Figure 1B and Table 1). This ALF-driven changes on the *M.tb* cell envelope are correlated with a significantly better control of *M.tb* intracellular growth by professional phagocytes *in vitro* by increasing phagosome-lysosome fusion, as well as long-term effects *in vivo* (Arcos et al., 2011, 2015; Moliva et al., 2019) (Table 1), suggesting a critical role of these first interactions between *M.tb* and host innate soluble proteins in determining infection and disease outcome. In fact, *M.tb* exposure to ALF, besides modifying the *M.tb* cell envelope, can also drive differential *M.tb* infection outcome in alveolar epithelial cells (ATs). In this regard, two distinct subsets of human ALFs are defined, the ones that upon exposure drove *M.tb* to replicate faster (H-ALF), and the ones that upon exposure slowed down *M.tb* replication (L-ALF) within ATs. This *M.tb* differential replication within ATs is further correlated with an altered human ALF composition and function, where people with H-ALF could be more susceptible to *M.tb* infection (Scordo et al., 2019) (Table 1).

**TABLE 1:** DS-*M.tb* and DR-*M.tb* interactions with the host at different stages of the infection.

Lung environments	DS-*M.tb*	DR-*M.tb*
**Initial *M.tb*–host interactions** ALF (e.g., hydrolases, surfactant proteins, etc.)	• Decrease of ManLAM and TDM in the cell envelope surface: increased P-L fusion in phagocytes (better control of *M.tb* intracellular growth). • Differential *M.tb* infection outcome in ATs after ALF exposure: H-ALF vs. L-ALF (based on ALF composition and functionality: host component). • Increased neutrophil’s capacity to eliminate ALF-exposed *M.tb* (increased P-L fusion and higher levels of TNF and IL-8, but reduced oxidative burst).	• Thicker cell envelope. • Presumably altered levels of fatty acids, TDM, TMM, glycerophospholipids, GLs, PDIM, PGL. • Presumably altered protein composition. • Presumably altered ALF-driven cell envelope modifications.
**Initial stages of infection** Macrophages, Neutrophils, ATs	• PAMPs in the *M.tb* cell envelope recognized by macrophages, DCs and other immune cells through different receptors. • Mannose-containing molecules in the cell envelope promote recognition and survival inside phagocytic cells. Host defense and immunomodulatory roles. • Efflux pumps correlated with development of drug resistance. • ManLAM, TDM, and PDIM levels associated with arrest of phagosomal acidification. • Virulence and pathogenesis correlated with the strain’s ability to invade ATs (protective intracellular niche).	• Increased expression of efflux pumps. • Reprogramming of host macrophage metabolism (bypassing the IL-1R1 pathway and inducing INF-β). • Remodeling of host transcriptional profiling through epigenetic manipulation. • Altered cytokine/chemokine production in macrophages. • Strains with low PDIM levels associated with low virulence. • Increased adhesion and invasion of ATs: successful dissemination. • Altered levels of CD3, CD4, NKT, CD4/CD8 ratio, TNF levels in serum: immune dysfunction.
**Latent *M.tb* infection Granuloma**	• Preference toward metabolic pathways using free fatty acids. • Dynamic remodeling of *M.tb* cell proteome and lipid metabolic networks. • Altered exporter function. • DS-*M.tb* becomes phenotypically drug-resistant (cell wall thickening). • Overexpression of efflux pumps.	• Transient drug-tolerance and permanent drug-resistance associated with trehalose-catalytic shift. • Similar phenotype as DS-*M.tb* in the granuloma environment.
**Reactivation/active TB disease**	• Increased production of mycolic acids, PDIM, SL-1, and PATs. • Biofilm formation in cavities (extracellular matrix of free mycolic acids).	• Currently unknown, although expected to have similarities to the interactions specifically described for DS-*M.tb* strains at this stage of the infection. It is at this stage when DR-*M.tb* is in cavities supposedly exposed to multiple drugs during DR-TB treatment and thus, DR-*M.tb* could also become resistant to these drugs (Sharma et al., 2020).

*DS-M.tb, drug-susceptible Mycobacterium tuberculosis; DR-M.tb, drug-resistant Mycobacterium tuberculosis; ALF, alveolar lining fluid; TDM, trehalose dimycolate; ATs, alveolar epithelial cells; H-ALF, high-ALF; L-ALF, low-ALF; TMM, trehalose monomycolate; GLs, glycolipids; PDIM, phthiocerol dimycocerosate; PGL, phenolic glycolipids; PAMPs, pathogen-associated molecular patterns; DCs, dendritic cells.*

Indeed, altered ALF composition and functionality in certain human populations such as the elder, HIV-infected, diabetic and/or individuals with underlying chronic lung diseases might be a contributing factor related to increased susceptibility to *M.tb* infection (Moliva et al., 2014, 2019; Bell and Noursadeghi, 2018; Segura-Cerda et al., 2019), demonstrating the protective and unique role of ALF in determining *M.tb* infection control and/or progression to disease. In this context, the elderly population is characterized by a pro-inflammatory lung environment, with increased protein oxidation levels in the ALF linked to decreased soluble innate immune function (Moliva et al., 2014, 2019). Thus, *M.tb* exposed to ALF obtained from elderly people and subsequently used to infect human macrophages shows altered trafficking (e.g., decreased phagosome-lysosome fusion) and increased *M.tb* intracellular growth (Moliva et al., 2019). This phenomenon is also observed *in vivo*, when elderly ALF exposed *M.tb* infected mice presented significantly higher bacterial burden and tissue damage in their lungs (Moliva et al., 2019). This increase in virulence when *M.tb* is exposed to elderly ALF is further linked to surfactant protein-D (SP-D) dysfunctionality in the lungs of elderly individuals (Moliva et al., 2019). In this context, polymorphisms in SP-D are linked to TB susceptibility (Azad et al., 2012; Hsieh et al., 2018). SP-D is shown to bind and cluster *M.tb* bacilli, influencing uptake by macrophages and intracellular killing by increased phagosome-lysosome fusion (Ferguson et al., 1999, 2006). These studies demonstrate the importance of the first *M.tb* interactions with soluble host innate immune components and the host ALF composition and functional status in dictating infection progression and disease outcome (Ferguson et al., 1999; Torrelles et al., 2008a). Still, the overall contribution of human ALF as an environmental pressure determinant in the development of DR-TB is a relatively unexplored research area. How *M.tb* changes its metabolism in response to these initial ALF interactions is still largely unknown, as well as which are the specific mycobacterial and host determinants that play a role in establishing a successful infection. Outcomes of *M.tb* infection after interacting with some ALF components [e.g., complement component 3 (C3), mannose binding lectin (MBL), SP-A, SP-D, etc., are described in detail elsewhere (reviewed in Torrelles et al., 2008a; Torrelles and Schlesinger, 2017; Garcia-Vilanova et al., 2019)].

The *M.tb* cell envelope surface composition plays a key role in defining the interactions between *M.tb* and its surroundings, with differences known to exist among *M.tb* clinical isolates (Torrelles et al., 2008b). Indeed, differential glycosylation patterns observed in membrane- and cell envelope-associated *M.tb* proteins, lipids and lipoglycans might contribute to virulence and phenotypic variability across *M.tb* lineages, including drug resistance development (Birhanu et al., 2019). Although little is known about the cell envelope composition of drug-resistant *M.tb*, some studies observed differences in cell envelope thickness between susceptible and resistant strains (Table 1). A study using electron transmission microscopy (TEM) showed differences of almost 2 nm and up to 4.6 nm in cell envelope thickness between MDR and XDR strains, respectively, when compared to susceptible isolates, with a denser peptidoglycan layer in XDR-*M.tb* strains (Velayati et al., 2009a) (Figure 1B). Further, XXDR-*M.tb* strains in exponential phase studied by atomic force microscopy present alterations in cell morphology (increased roughness and striations, with tubular extensions), probably induced by drug treatment; where a subpopulation of XXDR-*M.tb* bacteria (5–7%) had extraordinarily thick cell envelopes, independent of strain genotype (Velayati et al., 2010). Little information is provided about the drug-resistant strains tested in these studies, and it is still unknown if this thicker cell envelope is a general feature of all DR-*M.tb* strains; however, these microscopy studies suggest that the *M.tb* cell envelope composition is altered in these strains (Table 1).

Other studies attempted to analyze differences in lipid profiles between susceptible and DR-*M.tb* strains, as well as to study the role of surface lipids remodeling contributing to the emergence of MDR-*M.tb* phenotypes. Using a high-throughput mass spectrometry-based lipidomic approach, it is shown that MDR-*M.tb* strains have increased levels of free fatty acids, TDM, glycerophospholipids, altered glycerolipids, and unique and distinct lipid signatures when compared to drug-susceptible isolates (Pal et al., 2017) (Figure 1A and Table 1). Indeed, a potential link between low PDIM and PGL levels in the *M.tb* cell envelope with increased drug susceptibility has been reported using a *tesA* (encoding a type II thioesterase) mutant strain of *Mycobacterium marinum* (Chavadi et al., 2011). On the other hand, a recent study used two genetically distinct *M.tb* clonal pairs (laboratory and clinical drug-sensitive strains and their derived isoniazid-resistant (INHr) mutants) to determine specific *in vitro* changes related to the isoniazid-resistant phenotype through proteomic and lipidomic analyses (Nieto et al., 2018). These INHr isolates presented 26 proteins with altered levels, which were mainly associated with energy metabolism and respiration, but also with lipid metabolism, virulence, adaptation, and cell envelope remodeling. These strains presented activation of an alternative mycolic acid biosynthesis route, which is also observed in DR-*M.tb* strains from other lineages (Singh et al., 2015; Nieto et al., 2016b). Interestingly, of the two INHr mutants studied, only the clinical INHr isolate presented low levels of TDM, TMM (trehalose monomycolate) and PDIM, the latter associated with reduced virulence of this strain (Nieto et al., 2016a). This is in contrast with previous studies, indicating that *M.tb* genetic background may play and important role in determining the remodeling of the *M.tb* cell envelope protein and lipid constituents after acquisition of drug resistance (Table 1).

How differently DR-*M.tb* strains interact with the human alveolar environment is still unknown, and it is an active branch of research in our laboratory. In this context, during infection ALF soluble components could modify and shape differently the DR-*M.tb* cell envelope, as well as different DR-*M.tb* strains could have different metabolic responses to the alveolar environment, being an unexplored contributing factor in the development of drug resistance and/or persistence within the host (Table 1). Further investigation of a larger number of DR-*M.tb* isolates with resistance to different drugs is needed to support this hypothesis of a generally thickened and altered cell envelope and to determine interactions with ALF components.

### Active TB Disease: Interaction With Professional and Non-professional Phagocytes and Other Immune Cells in the Lung

After susceptible or DR*-M.tb* are bathed in immune soluble components of the ALF for an undetermined period of time, AMs are thought to be the first host cellular line of defense against *M.tb*, participating in the pathogen elimination directly or indirectly by activating host innate and adaptive immune responses (Figure 1C). AMs are defined as alternative activated, highly phagocytic cells with tightly regulated balance of pro-and anti-inflammatory responses that avoid the destruction of lung tissue due to excessive inflammation (Evren et al., 2020). *M.tb* has evolved to exploit AM resources as a strategy for host immune evasion and survival (Guirado et al., 2013).

Pathogen-associated molecular patterns (PAMPs) present in the *M.tb* cell envelope are recognized by macrophages and other phagocytic cells like lung submucosal and interstitial dendritic cells (DCs) directly or after being ALF-opsonized through an array of phagocytic receptors [e.g., C-type lectin receptors (CLRs), the mannose receptor (MR), Fc and complement receptors (CRs), DC-specific intercellular adhesion molecule-3-Grabbing non-integrin (DC-SIGN)], and signaling receptors [e.g., Toll-like receptors (TLRs)], among others (Leemans et al., 2003; Sasindran and Torrelles, 2011; Ishikawa et al., 2017; Rajaram et al., 2017; Lugo-Villarino et al., 2018) (Figure 1C and Table 1). After recognition and binding by a phagocytic receptor, the internalization of *M.tb* will initiate a series of trafficking and signaling cascades and the activation of numerous cellular processes such as *M.tb*-containing phagosome maturation, oxidative and inflammatory responses, antigen processing and presentation, autophagy, and cellular apoptosis, all essential phagocyte killing mechanisms and innate immune responses leading to clearance of *M.tb* infection (Mortaz et al., 2015) (Figure 1D). Although there are multiple scenarios of interaction with phagocytic cells via different receptors, it is unclear how *M.tb* uptake through these different phagocytic routes will affect disease progression, or if alternative routes lead to different outcomes.

Nevertheless, *M.tb* has learned to circumvent host defenses (Kang et al., 2005; Singh et al., 2009; Barczak et al., 2017; Cambier et al., 2017; Liu et al., 2018; Peterson et al., 2019), even to limit its exposure to sub-lethal concentrations of antimicrobial drugs potentially promoting the emergence of drug resistance (Wu et al., 2019). Indeed, it is shown that *M.tb* cell envelope surface mimics mammalian mannoproteins by having terminal mannose-containing molecules (e.g., ManLAM, higher-order PIMs, 19 kDa and 24 kDa mannoproteins, PstS-1, Apa, etc.). It is suggested that some strains of *M.tb* use these heavily mannosylated cell surface molecules to interact with lung surfactant proteins such as SP-A (Ragas et al., 2007), and phagocytic receptors including MR (Kang et al., 2005; Torrelles et al., 2006; Torrelles and Schlesinger, 2010; Esparza et al., 2015), Dectin-2 (Decout et al., 2018), and DC-SIGN (Pitarque et al., 2005; Tailleux et al., 2005; Driessen et al., 2009), providing a safe portal of entry for *M.tb* inside alveolar host cells without driving inflammation and limiting tissue pathology (Ehlers, 2010) (Table 1). In addition, collectins such as mannose-binding lectin (MBL) promote *M.tb* phagocytosis through bacterial opsonization by binding to ManLAM and PIMs, resulting in establishment of the infection (Denholm et al., 2010; Kalscheuer et al., 2019).

To date, multiple mycobacterial mannoproteins have been identified, with potential roles in virulence, cell invasion, evasion of host defense, and host immunomodulation, reviewed in Mehaffy et al. (2019), Deng et al. (2020), and Tonini et al. (2020). Many of these antigenic glycoproteins contain acyl groups and are defined as lipoglycoproteins (Becker and Sander, 2016). This is the case of the 19 kDa lipoglycoprotein LpqH, which has been shown to have multiple immunomodulatory roles such as: promoting the activation of neutrophils and CD4^+^ T cells, acting as an adhesin and promoting phagocytosis through lectin receptors, altering macrophage antigen presentation functions, or favoring *M.tb* immune evasion and dissemination by promoting TLR2-dependent macrophage apoptosis, among others (Parra et al., 2017). Another example is the 24 kDa lipoglycoprotein LprG, which binds and transports PIM and ManLAM to the cell surface of *M.tb*, acts as an inhibitor of MHC-II antigen processing in human macrophages *in vitro* (Gehring et al., 2004), although evidence suggests that LprG-reactive T cells are activated through TLR2 and glycosylation of specific MHC-II restriction molecules *in vivo* (Sieling et al., 2008). LprG is also associated with regulating TAG levels, *M.tb* growth rate and virulence in combination with the efflux pump Rv1410 (Martinot et al., 2016). Also, the 38 kDa mannosylated glycoprotein PstS-1 acts as adhesin by binding to the macrophage MR, promoting phagocytosis and intracellular survival (Esparza et al., 2015), and glycosylated lipoprotein SodC (Sartain and Belisle, 2009), a superoxide dismutase produced by *M.tb*, is known to act as a B-cell antigen and contribute to *M.tb* virulence, with a potential role in the defense against the oxidative burst produced *in vivo* inside macrophages (Piddington et al., 2001).

Other groups of glycoproteins involved in *M.tb*–host interactions are the ones belonging to the MCE family, MPT64, or Apa, among others, involved in colonization and invasion of host cells (Sonawane et al., 2012), as well as other proteins such as Rv0227c, HtrA-like serine protease Rv1223, TatA, GlnA1, and the disulfide oxidase DsbA-like enzyme Rv2969c, recently described as mannosylated (Tonini et al., 2020). In addition, GroEL2, a chaperone-like *M.tb* capsule-associated glycoprotein, is shown to contribute to the suboptimal antigen presentation during mycobacterial infection by modulating macrophage and DC proinflammatory responses (Georgieva et al., 2018), supporting the immunogenic role of *M.tb* cell envelope-associated glycoproteins during *M.tb* infection. Other strategies used by *M.tb* to counteract host defense mechanisms are the blockade of phagolysosome biogenesis and phagosomal acidification, where cell envelope components such as ManLAM and TDM among others, play important roles (Fratti et al., 2003; Vergne et al., 2004; Kang et al., 2005) (Table 1). A recent study also showed that *M.tb* surface protein Rv1468c binds to ubiquitin, triggering host xenophagy and promoting a controlled persistent intracellular infection while restricting host immune responses (Chai et al., 2019). Increased PIM expression is also reported during *in vitro* macrophage infection, although its involvement in *M.tb* virulence and intracellular survival is still unclear (Glass et al., 2017). The presence of DIM/PDIM lipids in the cell envelope is also associated with arrest of phagosomal acidification and macrophage death (Quigley et al., 2017).

Another important group of cell envelope glycoproteins are the ones associated with drug efflux pumps and mycobacterial cell wall permeability, with key implications in the development of drug resistance and *M.tb* persistence, especially when no genomic mutations are involved [reviewed in Singh et al. (2020)]. A clear example is the MmpL family, a group of inner membrane proteins associated with the transport of cell envelope lipids such as TMM, PDIM, sulfolipids, and TAG, among others (Melly and Purdy, 2019). Several MmpL proteins also act as drug efflux pumps, e.g., overexpression of MmpL5/S5 and MmpL7 increases mycobacterial resistance to clozafimine, bedaquiline, azole drugs, and isoniazid (Rodrigues et al., 2012; Hartkoorn et al., 2014). Indeed, antibiotic stress induces expression of efflux pumps in clinical *M.tb* strains (Gupta et al., 2010), which can contribute to the emergence of MDR-*M.tb* phenotypes. This is evidenced by the significantly increased expression of multiple efflux pumps, including Tap, among others, in MDR- and XDR-*M.tb* isolates when compared to drug-susceptible strains such as H_37_R_v_ (Li et al., 2015; Kardan-Yamchi et al., 2019; Liu et al., 2019) (Table 1). Conversely, the outer membrane channel protein CpnT that mediates efficient nutrient uptake during infection, can act as a major drug susceptibility determinant for *M.tb*, where mutations in *cpnT* appear to be associated with increased drug resistance in *M.tb* clinical isolates (Danilchanka et al., 2015). Other transporters involved in drug susceptibility/resistance have been identified in *M.tb* (da Silva et al., 2011; Singh et al., 2020). Overall, a systematic screening of cell surface-exposed lipids and glycoproteins and their functions is key for a better understanding of the pathogenesis and survival strategies adopted by DR-*M.tb* strains, including the development of drug-resistance *in vivo*.

At the end, the balance between macrophage capacity to control intracellular replication and *M.tb* ability to escape macrophage killing may dictate the outcome of the infection. In this delicate interplay, both macrophage phenotypic and functional heterogeneity, as well as *M.tb* genotypic and phenotypic differences displayed by the infecting strain will play important roles in disease progression (Guirado et al., 2013). In this regard, a distinct CD11c^+^ CD11b^+^ AM subpopulation with a unique inflammatory signature was shown to preferentially harbor *M.tb* during infection (Lafuse et al., 2019). Conversely, infection with different *M.tb* genotypes and strains induce different cytokine production and immune responses in *in vitro* and *in vivo* models (Lopez et al., 2003; Chacon-Salinas et al., 2005; Sousa et al., 2020). Indeed, there are more than 2,000 genes differentially expressed by macrophages during infection with a lineage 2 MDR-*M.tb* Beijing strain (Leisching et al., 2016). Thus, the biological properties and the metabolic status of infecting *M.tb* bacilli, as well as the surface composition of their cell envelope, will determine how *M.tb* interacts and activates AMs to respond to the infection (Torrelles et al., 2008b; Kalscheuer et al., 2019).

Some evidence indicate that DR-*M.tb* strains may have a different cell envelope thickness and composition than their counterparts, drug-susceptible strains (Velayati et al., 2009a, 2010; Singh et al., 2015; Nieto et al., 2016b, 2018). These potential differences in their cell envelope composition and thickness might be further accentuated by host ALF hydrolases and other host innate immune soluble components, differently impacting the interaction of these strains with AMs and subsequent infection progression. Indeed, MDR-*M.tb* isolates are described to present morphological differences in their cell envelope, that correlate with their selective advantage over other circulating *M.tb* isolates (Silva et al., 2013) (Table 1). Several high-throughput transcriptomic, proteomic and lipidomic approaches focused on the cell wall of *rpoB* mutant strains, showing upregulation of enzymes involved in the biosynthesis of PDIM (Bisson et al., 2012), decreased synthesis of fatty acids (du Preez and Loots, 2012), and altered levels of sulfoglycolipids and mycobactin (Lahiri et al., 2016). Other studies indicate that the *rpoB* mutation H526D is associated with altered cell wall physiology and resistance to cell-wall related stresses (Campodonico et al., 2018), and defined *rpoB* mutations as drivers of altered cytokine and chemokine production in macrophages (Ravan et al., 2019) (Table 1). Recently, MDR W-Beijing *M.tb* lineage 2 strains with rifampicin-conferring mutations in the *rpoB* gene were shown to overexpress cell envelope lipids such as PDIMs, bypassing the IL-1 receptor type I (IL-1R1) pathway associated with *M.tb* control and inducing INF-β that drives less effective aerobic glycolysis, ultimately reprogramming the metabolism of host macrophages (Howard et al., 2018; Howard and Khader, 2020) (Figure 1C and Table 1). Still, global effects of *rpoB* mutations on *M.tb* metabolism and cell envelope constitution, and subsequent interactions with host immune cells are still not well characterized, and further studies are needed to determine if the known effects are common to all DR-*M.tb* strains. Finally, there are some clinical evidences indicating that MDR-*M.tb* strains could be remodeling host transcriptional programming through epigenetic manipulation (Marimani et al., 2018; Crimi et al., 2020) (Table 1). Indeed, some XDR-*M.tb* strains seem to promote aberrant epigenetic modifications in macrophages, showing increased methylation levels of inflammatory genes in the TLR2 signaling pathway (Behrouzi et al., 2019).

Neutrophils are also key players in the host immune response against *M.tb*, being one of the first recruited innate effector cells to arrive at the infection site, found in large numbers in the lung during active TB disease (Eum et al., 2010) (Figure 1C). Upon *M.tb* contact, neutrophils can activate different intracellular and extracellular killing mechanisms to clear the infection, such as phagocytosis, production and release of reactive oxygen intermediates, and secretion of neutrophil extracellular traps (NETs) and granules containing hydrolytic enzymes and antimicrobial peptides (Kroon et al., 2018). Despite neutrophil’s protective role against *M.tb*, a regulation of how many neutrophils migrate to the infection site is important. Indeed, increased number of neutrophils in the alveolar space is associated with lung pathology during active TB due to excessive inflammation and tissue destruction, reviewed in detail elsewhere (Dallenga and Schaible, 2016; Muefong and Sutherland, 2020). Furthermore, neutrophil’s activity during *M.tb* infection is influenced by the human lung mucosa. In this regard, *M.tb* exposed to ALF enhanced the neutrophil’s innate capacity to recognize and kill *M.tb* intracellularly by enhancing phagosome-lysosome fusion events and producing higher levels of TNF and IL-8, while limiting excessive extracellular inflammatory responses and tissue damage by reducing oxidative burst, apoptosis, and degranulation (Arcos et al., 2015). Conversely, released *M.tb* cell envelope fragments after ALF exposure activate neutrophils, leading to an increased local oxidative response and the production of inflammatory cytokines, which regulate the activity of resting macrophages and thus, contribute to the control of *M.tb* infection (Arcos et al., 2017; Scordo et al., 2017) (Figure 1C). Thus, deciphering the effects of drug resistance in the *M.tb*–neutrophil interactions is a research area that needs further investigation.

ATs also play important roles in the early recognition and internalization of *M.tb* in the alveolar compartment (Scordo et al., 2016; Olmo-Fontanez and Torrelles, 2020). Upon *M.tb* recognition, ATs uptake *M.tb* and secrete cytokines, antimicrobial peptides, nitric oxide, surfactant proteins and other soluble components into the ALF, facilitating cell-to-cell crosstalk with other alveolar compartment cells such as AMs and neutrophils, and thus ATs are active players initiating the innate immune response (Garcia-Perez et al., 2003; Boggaram et al., 2013; Chuquimia et al., 2013; Reuschl et al., 2017; Gupta et al., 2018) (Figure 1C). Evidence supports that *M.tb* can replicate within ATs type II cells (ATII), localized inside late endosomal vesicles, and in some instances, ATs can process and present *M.tb* antigens via MHC class I, which are efficiently recognized by IFN-γ CD8^+^ T cells (Harriff et al., 2014), defining also ATs as contributors of the adaptive immune response initiation during *M.tb* infection and TB disease.

*Mycobacterium tuberculosis–*ATs interactions can occur during: the very first initial stages of primary infection when *M.tb* reaches the alveolar space or after *M.tb* is exposed to soluble host factors present in the lung mucosa (Scordo et al., 2019) (Figure 1C), following *M.tb* release from dying necrotic infected cells in advanced disease stages, and/or following release from granulomas during TB reactivation. The role of ATs might differ based on the stage of infection, although most of the studies are focused on initial *M.tb*–AT interactions and little is known about the other potential scenarios. Still, it is postulated that invasion of ATIIs could be beneficial for *M.tb*, as these cells constitute a protective intracellular niche optimal for undetected *M.tb* growth and replication, in part due to *M.tb*-containing late endosomes in ATs that fail to acidify, while evading recognition and clearance by professional alveolar phagocytes (Scordo et al., 2016). Indeed, virulence and pathogenesis of *M.tb* is correlated with their ability to infect ATs (McDonough and Kress, 1995), and differences in the *M.tb* cell envelope composition, pre-determined by the nature of host ALF, might be modulating *M.tb*–ATs interactions (Table 1). Supporting this, a *M.tb mce1* mutant strain accumulating mycolic acids in its cell envelope can bypass the TLR-2-mediated pro-inflammatory response in ATs (Sequeira et al., 2014). Studies also show that during infection, different *M.tb* genotypic strains induce different immune responses and gene expression patterns and activated pathways in ATs (Mvubu et al., 2016, 2018). Specifically, XDR-*M.tb* strains show increased adhesion and invasion of ATs compared to other genotypes, including drug-susceptible virulent *M.tb* H_37_R_v_ and attenuated H_37_R_a_ strains, suggesting that successful dissemination of DR-*M.tb* strains might be related to their interaction with the alveolar epithelium (Ashiru et al., 2010) (Table 1). We are just beginning to understand the role of ATs during *M.tb* infection and thus, it still remains unclear the differential adaptation of DR-*M.tb* strains to this alveolar cellular environment and its impact in *M.tb* infection and TB disease progression.

Differences in MDR-*M.tb* cell envelope and physiology may lead to different interactions with phagocytic and other immune cells, promoting characteristic host immune responses that might be different than the ones induced by susceptible *M.tb* strains. Indeed, several studies defined an array of different immune responses correlated to DR-*M.tb* strains. For example, a case-control study showed that MDR-TB patients had higher levels of CD3 and CD4 cells, as well as IgM, when compared to drug-susceptible TB patients (Sun et al., 2017). Another study showed that MDR-TB patient’s dendritic cells stimulated with MDR-*M.tb* antigens can mediate a significant higher production of IFN-γ by T cells when compared to DCs from uninfected individuals (Hadizadeh Tasbiti et al., 2018), although this study did not address how this MDR-*M.tb* induced immune responses compare to drug-susceptible *M.tb* stimulation. In this regard, another study showed that individuals infected with MDR-*M.tb* Haarlem strains had higher levels of IL-17^+^ IFN-γ^–^ CD4^+^ T cells through an IL-23 and TGF-β-dependent mechanisms when compared to latently TB-infected and uninfected individuals (Basile et al., 2017). Other host immune correlates have been associated with MDR- and XDR-TB patients when compared to both drug-susceptible TB and uninfected individuals: decreased levels of CD4, CD3/HLA-DR^+^ and Fas^+^ T cells, and increased levels of NKT and γδ T cells (Kiran et al., 2010); altered CD4/CD8 ratio and higher TNF levels in serum (Song et al., 2018); and low plasma concentrations of human neutrophil peptides (HNP1-3) (Zhu et al., 2011). T-regs might be also contributing to immune dysfunction and *M.tb* persistence in XDR-TB, although it is not clear whether this response is specific to XDR-TB or if it is actually associated with treatment failure independent of the strain’s drug resistance profile (Davids et al., 2018).

Overall, these data suggest that DR-*M.tb* strains induce different host immune responses (Table 1), and thus; there is a need of more robust clinical studies in order to establish whether the alteration of the host immune responses is an effect or a cause of DR-TB development.

### Latent TB: Granuloma Environment

After *M.tb* infection, host innate and adaptive immune responses are mounted, resulting in bacterial clearance, active TB disease or establishment of a latent *M.tb* infection (LTBI), the latter characterized by persistent *M.tb* bacilli contained inside complex structures called granulomas, a highly heterogeneous and dynamic cellular environment in the lung (Ehlers and Schaible, 2012; Guirado and Schlesinger, 2013) (Figure 1D). Depending of the granuloma stage (formation, maintenance, maturation, disruption leading to *M.tb* reactivation), these can be studied *in vitro* (Guirado et al., 2015) and *in vivo* using animal models such as mouse, Guinea pigs, rabbit and non-human primate models (NHPs) (Bucsan et al., 2019). Granulomas are composed of immune host cells including AMs, interstitial macrophages, foamy macrophages, monocytes, multi-nucleated giant cells, epithelial-like cells, DCs, NK cells, and neutrophils, surrounded by B and T cells, which tightly control *M.tb* replication and dissemination (Torrelles and Schlesinger, 2017; Muefong and Sutherland, 2020) (Figure 1D). Host determinants such as TNF, IL-6 and complement are important for cellular recruitment and granuloma maintenance (Dutta and Karakousis, 2014). Further, laser microdissection of individual granulomas, followed by confocal microscopy and proteomics, indicated that the granuloma’s center is a pro-inflammatory environment constituted of antimicrobial molecules, ROS and pro-inflammatory eicosanoids, whereas the tissue environment surrounding the granuloma possesses an anti-inflammatory profile (Marakalala et al., 2016).

Indeed, the granuloma environment has been characterized by low pH, hypoxia, oxidative stress, and nutrient starvation, where persisting bacilli are found in a metabolically adapted and non-replicating state (Keren et al., 2011; Eoh et al., 2017; Peterson et al., 2020). Using the *in vitro* granuloma model, *M.tb* is known to undergo metabolic changes, including having a preference toward using free fatty acids as energy source, in detriment of using carbohydrates (Guirado et al., 2015) (Table 1). Induction of the DosR regulon is also observed as a contributing factor in response to starvation and adaptation to other environmental stresses, suggesting a relevant role for the DosR regulon on the adaptation and persistence of *M.tb* within granulomas (Mehra et al., 2015; Zheng et al., 2020). Indeed, a proteome-wide study shows a highly dynamic remodeling of the *M.tb* proteome during exponential growth, dormancy and resuscitation (Table 1), where the stress-induced DosR regulon contributes 20% to cellular protein content during dormancy (Schubert et al., 2015). Remodeling of the *M.tb* cell envelope composition during dormancy seems to depend on phosphorylation by serine/threonine protein kinases (STPKs), which modulate lipid biosynthetic enzymes through altered exporter function (Table 1) (described in detail in Stokas et al., 2020).

Several *in vitro* models of hypoxia/nutrient depletion have also been used to study the cell wall of *M.tb* during forced dormancy [e.g., using media with no detergent or exogenous lipids, or *M.tb* cultured in the Hampshire model chemostat under gradual nutrient depletion in an oxygen-controlled environment (Galagan et al., 2013; Bacon et al., 2014)]. When compared to exponentially growing *M.tb*, dormant *M.tb* has a thicker cell envelope with increased content of TAGs, free mycolic acids, and lipoglycans, in detriment of having less TDMs, TMMs, and PDIMs, but keeping its infectivity and pathogenesis intact in a guinea pig model (Galagan et al., 2013; Bacon et al., 2014). Conversely, *in vitro* lipidomic studies using a modified Wayne’s model comparing exponentially grown *M.tb* in normoxic conditions vs. hypoxia-induced dormant *M.tb* (Gopinath et al., 2015) pointed a decrease in mycolic acids production and degradation of cell envelope-associated and cytoplasmic lipids during dormancy, which was attributed to a late onset of dormancy achieved in this model compared to previous studies. This lipid depletion was reversed to normal levels after bacterial re-aeration/resuscitation (Raghunandanan et al., 2019). Based on this model, reduction of mycolic acid content during dormancy could explain why *M.tb* is unable to elicit an appropriate host immune response and clearance during its dormancy state.

Granulomas also provide a protective environment for *M.tb* against anti-TB drugs, and delivery of drugs inside the core of the granuloma becomes challenging (Grobler et al., 2016). *M.tb* adaptation to the granuloma environment is also related to the thickening of the *M.tb* cell envelope (Cunningham and Spreadbury, 1998), where originally drug-susceptible *M.tb* bacillus become phenotypically drug-resistant (Deb et al., 2009; Rodriguez et al., 2014) or persisters (Figure 1D and Table 1). Further studies also showed *M.tb* overexpressing several efflux pumps within granulomas, contributing to *M.tb* drug tolerance during latency (Adams et al., 2011; Smith et al., 2013) (Table 1). In addition, stresses associated to the granuloma microenvironment (inflammation, hypoxia, starvation, among others) are correlated with increased *M.tb* mutation rates and the generation of drug resistance-conferring mutations in the *M.tb* genome, which have important implications in the emergence of DR-*M.tb* phenotypes *in vivo*. For example, host immune-mediated *M.tb* DNA damage suggests a potential role of error-prone DNA repair synthesis in the generation of chromosomally encoded drug resistance mutations, and emergence of persister populations might be associated to an altered metabolic state mediated by the (p)ppGpp alarmone system (Warner and Mizrahi, 2006). Actually, a study based on mathematical modeling estimated that the prevalence of latent MDR-TB is increasing worldwide, where one in every 83 individuals with latent TB is harboring an MDR-*M.tb* strain (Knight et al., 2019). Even though the increased cell envelope thickness observed in DR-*M.tb* strains resembles the appearance of dormant *M.tb* bacilli within granulomas, it is still unclear how DR-*M.tb* strains adapt to the granuloma environment, although transient drug tolerance and permanent drug resistance is associated with the bacterial trehalose-catalytic shift using an *M.tb* persister-like bacilli (PLB) model (Lee et al., 2019) (Table 1). Further research is needed in this area in order to develop novel anti-DR-TB strategies.

Finally, other type of host cells associated with granulomas are the mesenchymal stem cells. These host cells are reported to dampen the immune response to *M.tb* infection in tissues surrounding granulomas and thus, can provide a protective intracellular niche for *M.tb* replication during chronic stages of the infection, sheltering *M.tb* bacilli from anti-TB drugs and inflammatory cytokines, via increased PGE2 host signaling (Jain et al., 2020) (Figure 1D).

LTBI can persist for decades and thus, it is important to understand the balance between the host cells and *M.tb* creating this immune-controlled environment, especially in the case of DR-TB. When the host immune control is lost due to a variety of intrinsic (e.g., TNF blockade treatments) and external factors (e.g., malnutrition), which are still poorly understood, granulomas fail to contain *M.tb*, and latent infection reactivates and progresses to active TB disease (Figure 1E), with the formation of necrotic granulomas, extracellular *M.tb* growth and tissue destruction resulting in lung cavities and *M.tb* dissemination and/or escape to the upper airways to propagate the infection elsewhere. Decreased levels of TNF might be associated with this process, as well as T cell exhaustion or impaired functions (Lin et al., 2007; Garcia-Vilanova et al., 2019), although bacterial and other host factors leading to reactivation remain to be elucidated. It is suggested that during reactivation, *M.tb* increases the production of mycolic acids and other cell envelope components such as PDIM, SL-1, and pentaacyl trehaloses (PATs), increasing cell envelope hydrophobicity and subsequent transmission (Garcia-Vilanova et al., 2019) (Table 1). It is also shown that *M.tb* can form biofilms *in vitro* by creating a type of highly hydrophobic extracellular matrix composed of free mycolic acids (Ojha et al., 2008), which might shield the bacterium against host immune responses and high dose of TB drugs in necrotic areas such as cavities in the lungs (Orme, 2011, 2014), allowing bacterial persistence and transmission (Figure 1E and Table 1).

## Concluding Remarks

The emergence of DR-TB may be worsened by the current COVID-19 pandemic, exacerbating the global health crisis and undermining TB prevention and control strategies. The development of NGS technologies in the past 20 years has revolutionized our understanding of pathogen–host interactions in the infectious disease field, providing genome-wide information in an increasingly time- and cost-effective manner. Thus, whole-genome sequencing (WGS) has been used to gain better insight of *M.tb* biology and TB disease, including epidemiologic investigations, strain identification, TB transmission dynamics, pathogen–host interactions, and as a fast point-of-care (POC) diagnostic test (Wlodarska et al., 2015; Dookie et al., 2018; Brown et al., 2019) (Table 2). In this regard, numerous studies demonstrate the value of WGS in predicting DR-*M.tb* phenotypes from the *M.tb* genome sequenced directly from clinical specimens (e.g., sputum) (Groschel et al., 2018; Cohen et al., 2019b) (Table 2). Importantly, WGS has provided new insights into the complex underlying molecular mechanisms of *M.tb* drug resistance thanks to its nucleotide-level resolution (Gonzalez-Escalante et al., 2015; Yu et al., 2015; Coll et al., 2018) (Table 2). Of interest, studies of *M.tb* clinical isolates collected from the same patient at a single time point (parallel isolates) or at a different time points (serial isolates) during active TB disease progression and/or drug treatment, revealed within-host *M.tb* genomic diversity and microevolution events (O’Neill et al., 2015; Ley et al., 2019; Vargas et al., 2020). This observation was associated to *M.tb* drug resistance genes, lipid synthesis and regulation, and host innate immunity regulation. Thus, studying within-host *M.tb* diversity may assist in identifying novel adaptation strategies to drug and immune pressures in the different lung microenvironments (Nimmo et al., 2020).

**TABLE 2:** Next generation sequencing (NGS) strategies for drug-resistant *Mycobacterium tuberculosis.*

NGS approaches and applications	Advantages	Limitations
**WGS** • Species/strain identification. • Epidemiological studies. • TB diagnostics. • Molecular determinants of DR-*M.tb.*	• Nucleotide-level resolution: identification of single DR-*M.tb* conferring mutations. • Sequence directly from clinical samples (e.g., sputum). • Reveals *M.tb* genomic diversity and drug resistance microevolution events within the host.	• Only describe the genetic basis of DR-TB. • Limited information regarding DR-*M.tb* adaptation during disease progression and/or interactions with the host immune system.
**RNA-Seq** • Study DR-*M.tb* responses to drug treatment. • Study DR-*M.tb* metabolic/physiologic changes in different environment/conditions.	• Unbiased whole transcriptome approach. • High-throughput and relatively cost-effective. • Higher sensitivity and specificity compared to other gene expression approaches.	• Unable to distinguish DR-*M.tb* expression profiles of different strains in mixed infections. • Transcriptomic profile of either host or DR-*M.tb* in infected cells/tissues.
**Dual RNA-seq** • Study transcriptomic changes of both host and DR-*M.tb* simultaneously in infected cells/tissues.	• Global approach to study both host and DR-*M.tb*. • Establish causal host and DR-*M.tb* interactions: genome-wide association studies.	• Needs previous pathogen enrichment, and/or; • Increased sequencing depth to capture bacterial transcriptome (higher costs)
**scRNA-seq** • Study transcriptional responses of individual host cells or DR-*M.tb* bacillus during different physiological states (e.g., infection and disease progression). • Study DR-*M.tb* persister bacilli subpopulations during drug treatment. • Characterize individual responses in mixed infections.	• Unprecedented level of resolution and technological innovation (ability to barcode transcripts from a single cell). • Study host cell or DR-*M.tb* bacillus population heterogeneity. • Identify rare host cell or DR-*M.tb* bacillus populations. • Better understanding of tissue architecture.	• Lower throughput. • Requires effective isolation of viable *M.tb* bacilli or host cells. • Study either the host cell or *M.tb*, but mostly optimized for eukaryotes. • Need to overcome extra challenges in *M.tb* scRNA-seq [e.g., efficient lysis of the thick *M.tb* cell envelope, capture non-poly(A) mRNA]. • Complex bioinformatics analyses. • High sequencing costs.
**Dual scRNA-seq** • Study simultaneously host and pathogen at a single-cell resolution.	• Combined advantages of dual and scRNA-seq. • Define spatial-time relationships between host and pathogen. populations during different stages of the infection.	• Same as dual RNA-seq and scRNA-seq. • Difficult to extract enough bacterial information due to the low coverage and DR-*M.tb* bacilli:host ratio. • Complex interpretation of results.
**Single-cell multi-Omics** • Global understanding of complex DR-*M.tb* and host interactions through the integration of different “Omics” strategies, at a single-cell resolution.	• Systematic approach. • Dissect complex biological networks within a single-cell. • Integrate information from numerous “Omics.” • Highest level of information.	• Need for optimized and standardized protocols and bioinformatics approaches. • Difficult to integrate and interpret the data. • Single-cell resolution not easily achievable for some “Omics” approaches.

*DR-M.tb, drug-resistant Mycobacterium tuberculosis; scRNA-seq, single-cell RNA sequencing; TB, tuberculosis; WGS, whole-genome sequencing.*

Importantly, during infection, DR-*M.tb* encounters very different human lung microenvironments, modifying its metabolism to adapt and persist in the human host. Evidence suggests that the initial *M.tb*–host interactions in the alveolar space, such as ALF exposure, play a decisive role in the establishment of *M.tb* infection. Indeed, ALF-derived changes in the *M.tb* cell envelope drive interactions with host innate immune cells, modulating the immune response and infection outcome. Although only a few studies have focused on the *in vitro* and *in vivo* pathogenesis and adaptation of DR-*M.tb* strains, a differential host response to DR-*M.tb* when compared to drug-susceptible strains is reported, which might be due, in part, to an altered *M.tb* envelope composition leading to *M.tb* clearance, active TB disease or latent *M.tb* infection.

In order to develop new strategies to combat the emergence of DR-*M.tb* clinical isolates, it is imperative to decipher how DR-*M.tb* navigates through the different lung tissue microenvironments, adapting its metabolic status to persist. Transcriptomic approaches allow us to better understand the mode of action of particular drugs (Table 2), as well as mechanisms of drug tolerance and, in particular, those associated to bacterial metabolic/physiologic changes and to the host immune response, reviewed in detail elsewhere (Liu et al., 2016; Singhania et al., 2018; Briffotaux et al., 2019; Li et al., 2020). In the last decade, dual RNA sequencing has been investigated as an unbiased and global strategy to simultaneously characterize the transcriptional profiling of host and pathogen in infected cells/tissues (Avraham et al., 2016; Westermann et al., 2016, 2017) (Table 2). In this regard, dual RNA-Seq of *M. bovis* BCG-infected macrophages showed upregulation of mycobacterial cholesterol degradation and iron acquisition pathways, and recycling of mycolic acids, while host cells upregulated *de novo* cholesterol biosynthesis likely to compensate for the loss of this metabolite due to bacterial catabolism (Rienksma et al., 2015). Further, dual RNA-seq in *M.tb* infected macrophages isolated from mice show that the growth advantage of *M.tb* in AMs compared to interstitial macrophages (IMs) is a direct consequence of the metabolic interplay between *M.tb* and AMs (Pisu et al., 2020). A similar strategy could be applied to study how DR-*M.tb* strains adapt to the lung environment in comparison to drug-susceptible *M.tb* strains, and clearly define transcriptional profiles during infection that could be used in DR-TB diagnostics and/or therapy.

Single-cell RNA sequencing (scRNA-seq) has also gained popularity in recent years as a means to study individual transcriptional responses of different host cell populations during altered physiological states (e.g., infection) at an unprecedented level of resolution (Hosokawa et al., 2017; Kolodziejczyk and Lonnberg, 2018; Vegh and Haniffa, 2018; Hashimoto et al., 2019) (Table 2). Thanks to its ability to amplify hundreds to thousands of individual cells in a single preparation, it allows the identification of rare cell populations that otherwise might go undetected in bulk RNA-seq (Tirosh et al., 2016). However, only a few studies have been conducted in the TB field (Gierahn et al., 2017), due to numerous challenges associated with the single-cell isolation and cell wall disruption of *M.tb*, capturing *M.tb* non-polyadenylated mRNA, and amplifying the low amount of prokaryotic starting material [reviewed in detail (Chen et al., 2017; Zhang et al., 2018)] (Table 2). Several methods are being developed in order to overcome these microbial scRNA-seq issues (Sheng et al., 2017; Blattman et al., 2020; Imdahl et al., 2020). In this context, scRNA-seq would be critical to study *M.tb* persisters within granulomas, where a small fraction of the total bacterial population is able to survive drug exposure and killing, likely due to different metabolic/physiological state leading to DR-*M.tb*.

Paired dual scRNA-seq, which combines the dual transcriptional profiling of host and pathogen in infected tissues with the high resolution of single-cell analysis, represents the next level in the study of complex host–pathogen interactions (reviewed in Penaranda and Hung, 2019) (Table 2). To date, only one study has attempted to simultaneously characterize the transcriptomes of both host and pathogen at a single-cell level in macrophages infected with *Salmonella typhimurium* (Avital et al., 2017), although it was difficult to extract meaningful biological information due to the low bacterial coverage, plus interpretation of results becomes increasingly complex (Table 2). Although there is still much to accomplish in this area, this methodology will become invaluable to study the adaptive capabilities of *M.tb* to drug treatments, as well as to study *M.tb*-ALF and *M.tb*-host cell responses during infection. We would be able to respond questions such as why during the same infection some *M.tb* bacterial subpopulations enter drug tolerant states during treatment and others don’t, why some *M.tb* bacilli but not others are cleared by the same host cell type during infection, and/or what is the spatial-time relationship between *M.tb* and the host cell defining the infection outcome.

Ultimately, we expect that single-cell multimodal ‘Omics’ approaches will be the future for dissecting the complex biological networks within each cell, providing a systematic and global picture by analyzing and combining multiple modalities such as genomics, transcriptomics, epigenomics, proteomics, lipidomics, and glycomics, among others (Khan et al., 2019; Zhu et al., 2020) (Table 2). Indeed, metabolomics and dual RNA-seq data from *M.tb*-infected macrophages were collected using a multi-omics approach to identify metabolic sub-networks regulated during early *M.tb* infection, showing that *M.tb* consumes up to 33 different host-derived substrates to establish its intracellular niche (Zimmermann et al., 2017).

These novel NGS strategies, including scRNA-seq, dual RNA-seq, and single-cell multi-Omics could provide systematic and global approaches to fully understand the complex DR *M.tb*-host interplay and dynamics during the different stages of the infection, and to uncover regulatory networks critical for DR-*M.tb* survival in the host that could potentially be exploited as novel anti-DR-TB strategies.

## Author Contributions

AA-G, JIG, and JBT made substantial contributions to the conception, writing, and editing of this review. All authors contributed to the article and approved the submitted version.

## Conflict of Interest

The authors declare that the research was conducted in the absence of any commercial or financial relationships that could be construed as a potential conflict of interest.
